# The Effect of Electrospun Gelatin Fibers Alignment on Schwann Cell and Axon Behavior and Organization in the Perspective of Artificial Nerve Design

**DOI:** 10.3390/ijms160612925

**Published:** 2015-06-08

**Authors:** Sara Gnavi, Benedetta Elena Fornasari, Chiara Tonda-Turo, Rossella Laurano, Marco Zanetti, Gianluca Ciardelli, Stefano Geuna

**Affiliations:** 1 Department of Clinical and Biological Sciences, University of Torino, Orbassano 10043, Italy; E-Mails: sara.gnavi@unito.it (S.G.); benedettaelena.fornasari@unito.it (B.E.F.); 2 Neuroscience Institute of the Cavalieri-Ottolenghi Foundation, University of Torino, Orbassano 10043, Italy; 3 Department of Mechanical and Aerospace Engineering, Politecnico of Torino, Torino 10100, Italy; E-Mails: chiara.tondaturo@polito.it (C.T.-T.); rossella.laurano@icloud.com (R.L.); 4 Nanostructured Interfaces and Surfaces, Department of Chemistry, University of Torino, Torino 10100, Italy; E-Mail: marco.zanetti@unito.it; 5 Department for Materials and Devices of the National Research Council, Institute for the Cehmical and Physical Processes (CNR-IPCF UOS), Pisa 56124, Italy; E-Mail: gianluca.ciardelli@polito.it

**Keywords:** peripheral nerve injury, artificial nerve organs, gelatin nano-fibers, electrospinning, aligned fibers

## Abstract

Electrospun fibrous substrates mimicking extracellular matrices can be prepared by electrospinning, yielding aligned fibrous matrices as internal fillers to manufacture artificial nerves. Gelatin aligned nano-fibers were prepared by electrospinning after tuning the collector rotation speed. The effect of alignment on cell adhesion and proliferation was tested *in vitro* using primary cultures, the Schwann cell line, RT4-D6P2T, and the sensory neuron-like cell line, 50B11. Cell adhesion and proliferation were assessed by quantifying at several time-points. Aligned nano-fibers reduced adhesion and proliferation rate compared with random fibers. Schwann cell morphology and organization were investigated by immunostaining of the cytoskeleton. Cells were elongated with their longitudinal body parallel to the aligned fibers. B5011 neuron-like cells were aligned and had parallel axon growth when cultured on the aligned gelatin fibers. The data show that the alignment of electrospun gelatin fibers can modulate Schwann cells and axon organization *in vitro*, suggesting that this substrate shows promise as an internal filler for the design of artificial nerves for peripheral nerve reconstruction.

## 1. Introduction

Peripheral nerve injury following trauma may lead to a substantial loss of nerve tissue, and long defect formation between the proximal and the distal nerve stump, rendering surgical intervention necessary. Currently, the gold standard for nerve repair involves bridging the nerve gap by using an autologous nerve graft taken from another part of the body. Because autograft techniques have several disadvantages, the use of artificial nerve organs composed of biomaterial may be the ideal choice among other options [1,2,3,4,5].

Numerous substances, both of synthetic or natural origin, have been used to fabricate artificial nerve conduits [3,6,7,8,9]. Synthetic, non-degradable polymers, such as silicone, and synthetic biodegradable polymers, such as poly(lactic-*co*-glycolic acid) (PLGA), poly-ε-caprolactone (PCL), poly-l-lactic acid (PLLA) and conductive polymers (polypyrrole, polyaniline), have been used for nerve injury repair [10,11,12,13,14]. More recently, several natural biodegradable polymers, such as collagen, chitosan, alginate, elastin, silk, fibrins and gelatin have been used in tissue engineering due to their bioactivity, biocompatibility, low toxicity, tunable mechanical properties and degradation kinetics [3,7,8,14,15]. Natural biomaterials have been manipulated to obtain biomimetic materials, particularly the topography and the 3-D conformation being adjusted, to simulate extracellular matrix (ECM) [2,16,17,18].

Conduits that are Food and Drug Administration (FDA) and European Commission approved consist of biodegradable materials, among them Neurotube™, Neura-Gen™ and Neurolac tubes made of poly(glycolide) (PGA), collagen and poly(dl-lactide-ε-caprolactone), respectively [2,3,19,20].

Collagen is the major connective tissue protein that is widely dispersed in the ECM of the peripheral nervous system (PNS) [21,22]. Over the past decades, collagen-based biomaterials have been widely used in tissue engineering [23]. Collagen is a biodegradable, biocompatible, highly versatile and readily available polymer. Despite these advantages, it is hard to sterilize without altering its native structure [23,24]. Moreover, the use of collagen in the construction of artificial scaffolds might cause adverse immune responses [23].

Gelatin may be used as an alternative to collagen in artificial organ preparation [25,26]. Gelatin is produced by thermal denaturation or physical and chemical degradation of collagen. In comparison to collagen, gelatin has many advantages: it is biocompatible, biodegradable and does not induce immune rejection problems, maintaining molecular cues that may regulate cell behavior [25,26]. Cross-linking may also be used to modulate the mechanical, chemical and topographic properties of gelatin [25,27,28].

Both collagen and gelatin have been used in the preparation of different internal filler in artificial nerve conduit manufacturing, among them hydrogels and fibers [2,27,28,29,30,31]. Collagen is included as a scaffold in polycaprolactone tubular prostheses, resulting in stimulation of nerve regeneration in the rat sciatic nerve model [32].

A thermosensitive collagen hydrogel mimicking ECM has been used as filler of poly-l-lactic acid scaffolds involving bone marrow mesenchymal stem cells [33]. Collagen has also been used in an aligned collagen-glycosaminoglycan matrix preparation, mimicking Schwann cells (SCs) basal lamina that permits SC migration and repopulation [34]. Oriented collagen fibers are included as fillers of collagen tubes, providing a guide for regenerating axons in an orientated manner to the distal nerve segment in a rat sciatic nerve transaction model [35]. Finally, the use of Revolnerv^®^ collagen tubes for palmar digital nerve repair in human patients has been reported; a six-month follow-up showed that this did not improve regeneration in comparison to an uncoated direct suture [36].

Gelatin is now being used to prepare electrospun fibers [27,28,29] and hydrogel [31,37,38] that have high biocompatibility towards SCs and axons. Cross-linked gelatin combined with ECM components and neurotrophins is a promising scaffold for SC and Dorsal Root Ganglia (DRG) [39]. Gelatin can also increase the biocompatibility of scaffolds used in neural tissue engineering [40].

Fibers for internal fillers in artificial nerve conduits can be prepared through an electrospinning technique [41,42,43]. Several parameters can be adjusted to produce random or aligned fibers, these being classified into solution parameters (*i.e*., concentration, molecular weight, solvents and polymer type), solution properties (viscosity, surface tension and conductivity), process parameters (*i.e*., applied voltage, flow rate, collectors type and tip to collector distance), and ambient parameters [41]. Fiber alignment is mainly determined by the type of collector (flat or cylindrical) and its rotation speed [41,42]. The electrospun fibers produced by these methods may affect cell adhesion, morphology, proliferation and differentiation [16,17,18]. Corrugated round fiber topography has given better adhesion and proliferation of C2C12 cells in comparison to solid round fibers [44].

Human embryonic stem cell-derived neural precursors seeded on polycaprolactone (PCL) aligned fibers showed an elongated morphology with their longitudinal axis parallel to the direction of PCL fibers [45]. DRG cultured on PCL fibers extend their neurites without specific directionality when cultured on random fibers. On the other hand, the neurites grew along the long axis of the fibers cultured on an aligned parallel fiber matrix [46]. Aligned and random PCL/gelatine nano-fibers promote Schwann cell adhesion and growth in comparison to PCL nano-fibers [40]; both PCL/gelatin and PCL aligned fibers oriented cells along the longitudinal direction of the fibers [40]. PCL aligned nano-fibers coated with GRGDS and YIGSR peptide act as guides, increasing actin filament alignment in Schwann cells [47]. Finally, poly( 3-hydroxybutyrate) aligned fibers have been used to differentiate PC12 cells; cultured on aligned fibers these cells had highly aligned and longer neurites in comparison to those on randomly oriented fibers [48].

We chose gelatin to produce random and aligned electrospun nano-fibers by the electrospinning technique. The influence of the different electrospun nano-fibers topography and 3D-structure of several *in vitro* cell models has been studied. Since bands of Büngner formation by SCs and axons regrowth are the key elements in nerve regeneration, the RT4-D6P2T line, primary SC cultures and the neuron-like 50B11 cell line were used. Particularly, RT4-D6P2T cell line is an immortalized Schwann cell line derived from a *N*-ethyl-*N*-nitrosourea (ENU) induced rat peripheral neurotumor. Primary SC cultures were obtained from rat sciatic nerves. The 50B11 cell line is an immortalized rat DRG sensory neuron cell line that can be induced to differentiate *in vitro.*

## 2. Results

### 2.1. Influence of Mandrel Collector Speed on the Alignment of the Nano-Fibers

**Figure 1:**
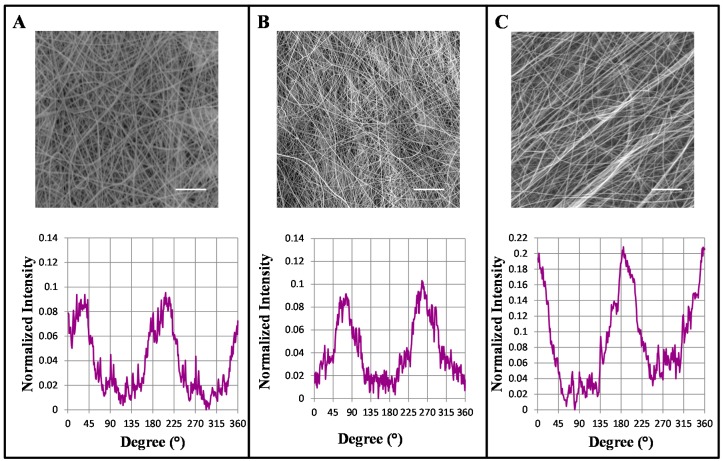
Scanning electron microscopy (SEM) micrographs and 2D fast fourier transform (FTT) analysis of nano-fibers collected using rotating mandrel rates of 300 (**A**), 1200 (**B**) and 2400 (**C**) rpm. Scale bars: 10 µm.

Randomly oriented fibers with an average fiber dimension of 300 nm were fabricated as previously described [28]. Aligned GL/PEO_GPTMS (gelatin/polyethylene-oxide/(3-Glycidoxypropyl)methyldiethoxysilane) nano-fibers were made using a rotating mandrel collector, and its rotation was varied from 0 to 2400 rpm to analyze the influence of this parameter on fiber alignment. FFT (2D Fast Fourier Transform) analysis of the SEM (Scanning Electron Microscopy) images was used to quantitatively analyze the degree of the GL based nano-fibers alignment. In FFT analysis, a graphical plot of frequency distribution was generated by summing the pixel intensities along the radius of the FFT output image obtained from the original SEM image. For a rotation of 2400 rpm, two sharp peaks were observed at a distance of ~180°, confirming the morphological data that showed a large number of fibers aligned in a preferential direction (Figure 1C). On the other hand, rotating at ~300 rpm produced no fiber orientation, confirmed by SEM image and FTT analysis (Figure 1A). Nanofibers size was measured at different rotating mandrel speeds showing a slight reduction in the fiber average diameters when the speed is increased. The measured diameters were 204 ± 48 nm for aligned nanofibers using a rotating speed of 2400 rpm, 238.9 ± 74 nm for 1200 rpm and 254.7 ± 68.5 nm for 300 rpm. No significant differences in fiber size were observed from fibers collected on plane or rotating collector. Nanofibers obtained using a rotating speed of 2400 rpm were used for cell test, as they showed a high degree of alignment on a preferential direction (as confirmed by FFT and SEM analysis)

### 2.2. Aligning Gelatin Nano-Fibers Decreased the Number of Adherent Schwann Cells

**Figure 2:**
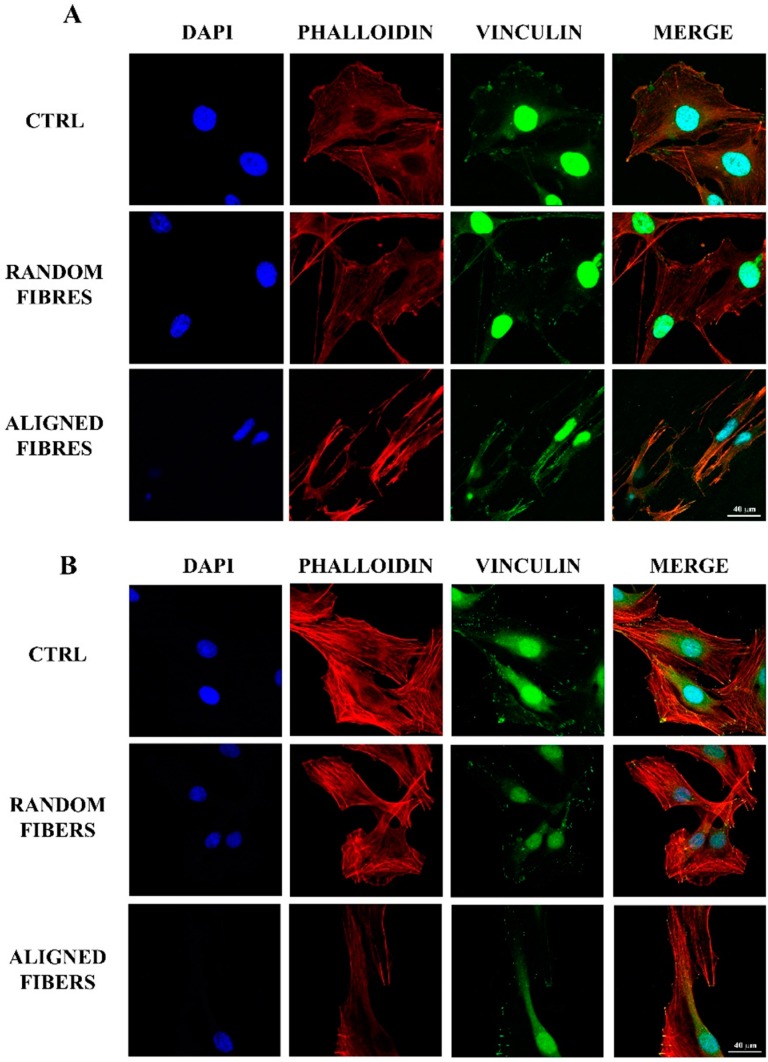

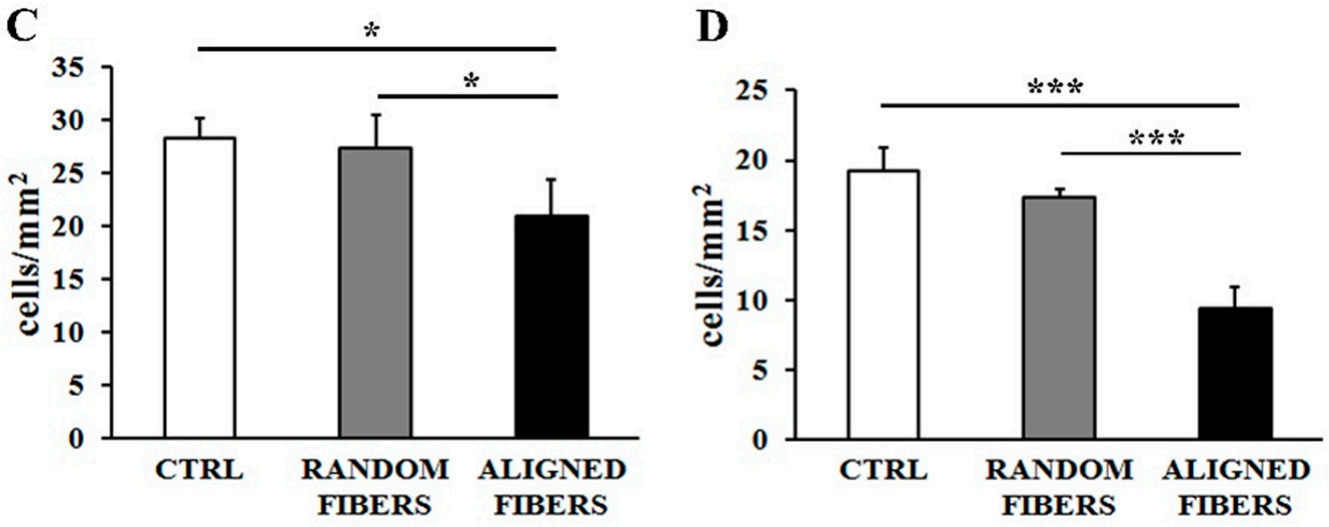
Adhesion assay: Confocal images (63× magnification) after DAPI (blue), tetramethylrhodamine (TRITC)-conjugated phalloidin (red) and vinculin (green) staining of RT4-D6P2T (**A**) and primary SC (**B**) on poly-l-lysine coated coverslips (control condition), random fibers and aligned fibers 3 h after seeding. Scale bar: 40 μm; RT4-D6P2T (**C**) and primary SC (**D**) cell numbers were expressed as cells/mm^2^ ± standard error of the mean (SEM). Statistical analysis was carried out using one-way ANOVA. Asterisks refer to significant statistical difference with * *p* ≤ 0.05 and *** *p* ≤ 0.001.

RT4-D6P2T and primary SC cultures were seeded on control condition (polylysine coated coverslips), gelatin random fibers and aligned fibers. After 3 h, the adherent cells were counted and their morphology examined. Figure 2 shows the effect of fiber alignment on cell adhesion and morphology. The alignment of gelatin electrospun fibers affected the number of adherent cells for RT4-D6P2T (*p* < 0.05) (Figure 2C) and primary SC (*p* < 0.001) (Figure 2D) cultures. When seeded on aligned fibers, there was less adhesion than under control conditions or random fibers. Both RT4-D6P2T and primary SC had high actin cytoskeleton organization and many focal adhesion points under all conditions tested. Its staining showed that both RT4-D6P2T and primary SC cultured on aligned fibers had elongated actin fibers compared to the control condition and random fibers (Figure 2A). Finally, cells had an elongated morphology with their longitudinal axis parallel to the direction of the aligned gelatin nano-fibers (Figure 2A).

### 2.3. Aligning Gelatin Nano-Fibers Reduced Schwann Cell Proliferation Rate

**Figure 3:**
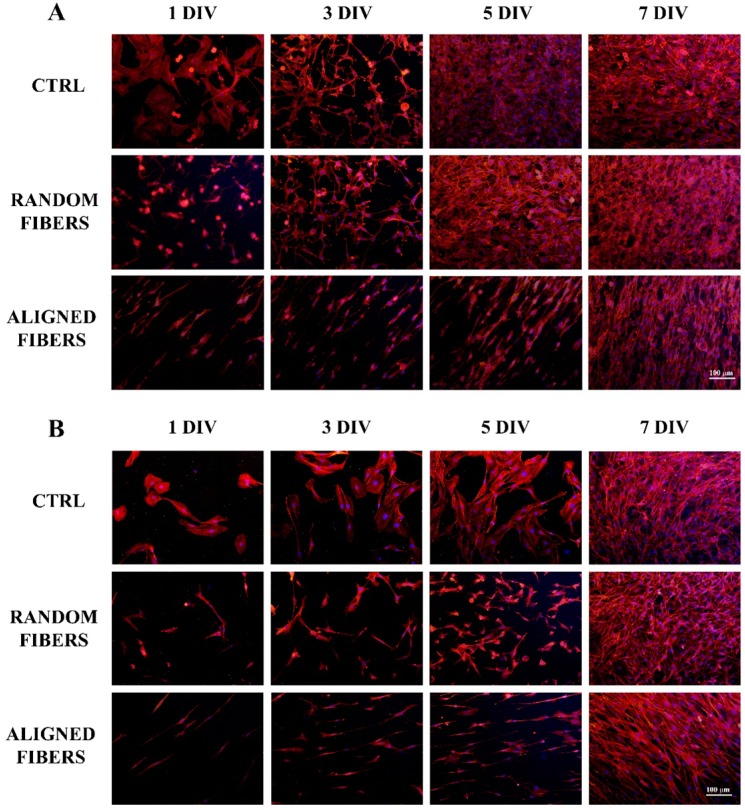

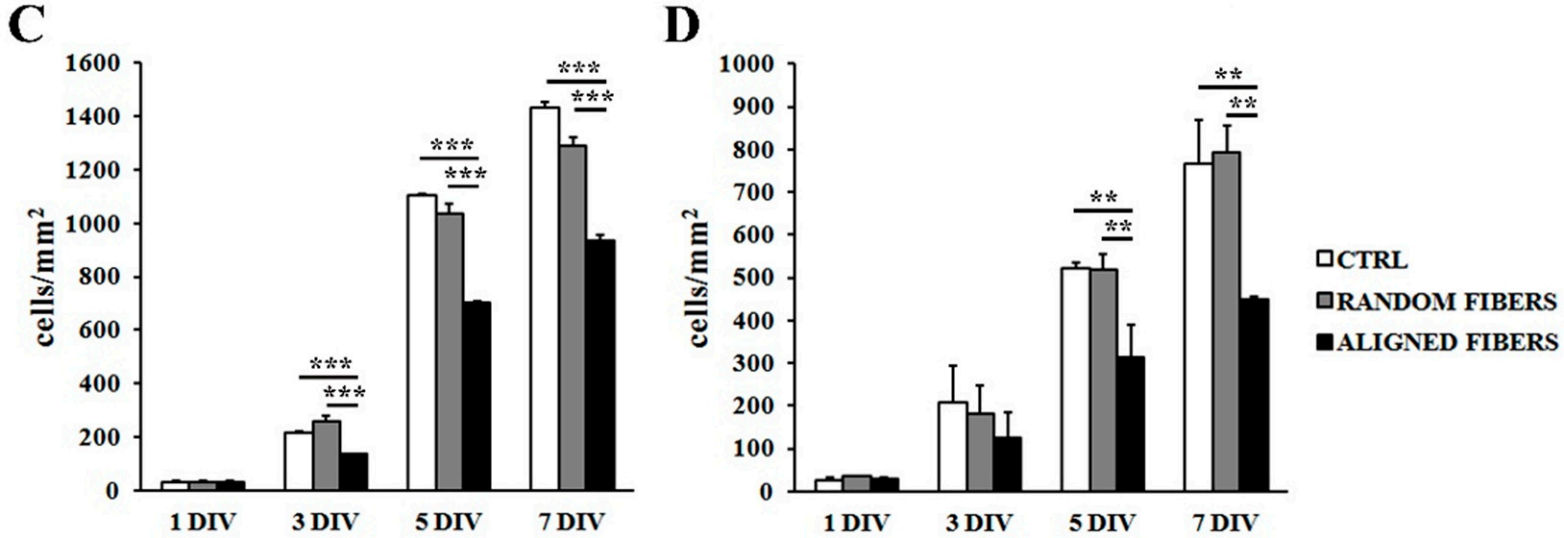
Proliferation assay: Fluorescent images (20× magnification) after DAPI (blue) and phalloidin (red) staining of RT4-D6P2T (**A**) and primary SC (**B**) on poly-l-lysine coated coverslips (control condition), random fibers and aligned fibers after 1, 3, 5, and 7 DIV (days *in vitro*) after seeding. Scale bar: 100 μm; RT4-D6P2T (**C**) and primary SC (**D**) cell number is expressed as cells/mm^2^ ± standard error of the mean (SEM). Asterisks refer to significant statistical difference with ** *p* ≤ 0.01 and *** *p* ≤ 0.001.

After three (*p* < 0.001), five (*p* < 0.001) and seven (*p* < 0.001) days, RT4-D6P2T cells proliferated more slowly when seeded on aligned fibers than under control conditions or random fibers (Figure 3C). After five (*p* < 0.01) and seven (*p* < 0.01) days, primary SC also had slower proliferation rates on aligned fibers than under control conditions or random fibers (Figure 3D). Both RT4-D6P2T and primary SC cells reached confluence under all conditions tested.

Primary SC staining of actin showed normal spread morphology at all time-points, consistent with the adhesion assay data (Figure 3B), similar results being obtained with RT4-D6P2T cells (Figure 3A). Both RT4-D6P2T and primary SC cultured on aligned fibers had elongated actin fibers in comparison to control condition and random fibers at all time-points. Cells are organized in aligned bands with their longitudinal axis parallel to the direction of the aligned gelatin nano-fibers (Figure 3A,B).

**Figure 4:**
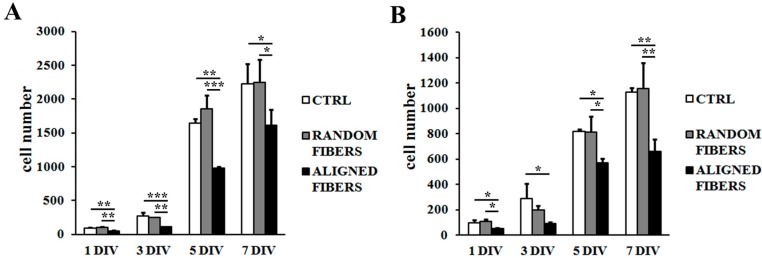
MTT assay: RT4-D6P2T (**A**) and primary SC (**B**) were seeded on poly-l-lysine coated coverslips (control condition), random fibers and aligned fibers. 1, 3, 5, and 7 DIV (days *in vitro*) after seeding, cell viability was quantified. Asterisks refer to significant statistical difference with * *p* ≤ 0.05, ** *p* ≤ 0.01 and *** *p* ≤ 0.001.

Finally, aligned gelatin fibers reduced Schwann cell proliferation rate after one, three, five, and seven days (Figure 4). The MTT assay confirmed that Schwann cell were highly viable with good vitality on both random and aligned fibers, indicating good biocompatibility of gelatin electrospun fibers.

### 2.4. Aligning Gelatin Nano-Fibers Resulted in Neurites Alignment

**Figure 5:**
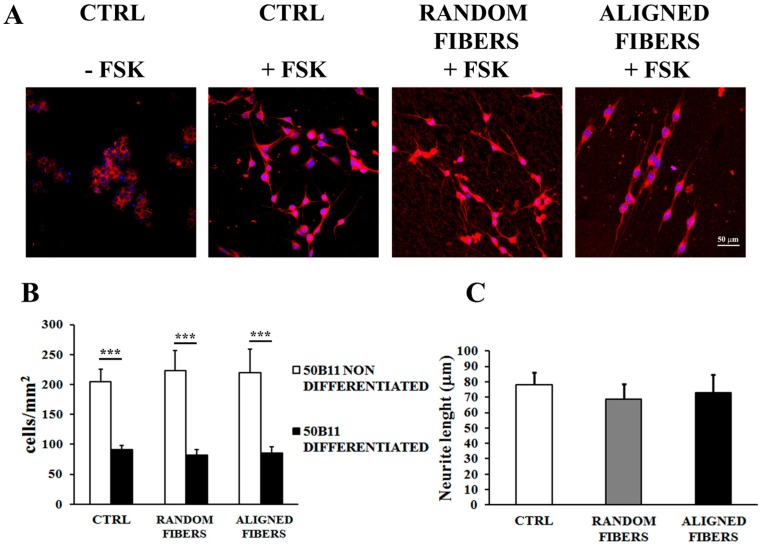
50B11 differentiation: Confocal images (40× magnification) after DAPI (blue) and β-tubulin (red) staining of B5011 seeded on poly-l-lysine coated coverslips (control condition), random fibers and aligned fibers 24 h after forskolin treatment. Scale bar: 50 μm. (**A**) 50B11 cell number is expressed as cells/mm^2^ ± standard error of the mean (SEM) (**B**); Neurite length is expressed in μm (**C**); Asterisks refer to significant statistical difference with *** *p* ≤ 0.001.

50B11 were seeded under control condition, and also on aligned and random fibers. After 24 h, the adherent cells were counted and their morphology examined by β-tubulin and DAPI staining. Addition of 75 μM forskolin resulted in 50B11 differentiation under all conditions tested (Figure 5). The alignment of gelatin electrospun fibers did not affect the number of adherent 50B11 cells (Figure 5B). After forskolin, 50B11 cells stopped proliferating and started differentiating, resulting in a reduced cell number (*p* < 0.001) compared with non-treated conditions (Figure 5B). Immunostaining of β-tubulin showed that 50B11 cell maintain their ability to differentiate on gelatin electrospun fibers. Confocal images showed that aligned fibers made neurites align in parallel to the direction of the fibers. Neurites growth on aligned fibers was similar to random fibers and control condition (Figure 5A). There were no differences in 50B11 neurites length under all conditions tested (Figure 5C).

## 3. Discussion

Interest in developing artificial nerve conduits composed of internal filler mimicking the extracellular tissue matrix has been increasing in recent years [2,4,49].

Many synthetic and natural materials have been used in the fabrication of artificial nerve conduits [3,6,7,8,9]. Initially synthetic non-degradable polymers, such as silicone, have been used to enhance nerve regeneration, even though they had several disadvantages for long-term recovery [10,11,12,13,14]. More recently, several natural bio-degradable-polymers, such as collagen [7,24,34], chitosan [15,50] and gelatin [25,26,29,31] have been used in tissue engineering applications due to their biocompatibility, mechanical and degradation properties [3,7,8,14,15]. These natural biomaterials have been used to obtain biomimetic materials displaying topography and the three-dimensional conformation similar to the ECM [2,16,17,18].

Since collagen, the main component of ECM, has some disadvantages such as adverse immune response induction [21,22,23,24], gelatin may be a suitable alternative to be used in the construction of artificial nerve for tissue engineering applications [6,7,25,27,28,29,31,40].

The aim of this study was understand: (i) the effect of gelatin fibers on SC and neuron viability; and (ii) the influence of electrospun nano-fibers alignment on Schwann cell adhesion, morphology, proliferation and axonal growth. Since SC and neurons play an important role in peripheral nerve regeneration processes [2,3,30], we used RT4-D6P2T SC line, primary SC culture and neuron-like 50B11 cell line to perform *in vitro* tests. In order to understand if the alignment of gelatin electrospun nano-fibers modulates SC organization and axonal growth PLL coated-coverslip and random nano-fibers have been used as control condition.

### 3.1. Increasing Mandrel Collector Speed Rotation Resulted in Fibers Alignment

We were previously successful in using an electrospinning technique to spin water gelatin solution to prepare random nano- and microfibers [27,28]. The use of aqueous solution for fibers preparation is known to reduce the risk of gelatin denaturation and nano-fibers cytotoxicity compared to organic solvents and acidic solutions. Homogenous nano-fibers with diameters of 200–300 nm were made and successfully aligned, using a rotating collector at 2400 rpm speed.

### 3.2. Fibers Alignment Reduced Schwann Cells Adhesion and Proliferation but Enhanced the Alignment of Schwann Cells Actin Filaments

In regards to cell adhesion, the reduced level in RT4-D6P2T and primary SC seeded on aligned nano-fibers may be due to the different fiber topography with which fewer focal adhesion points compared to random fibers [16,51]. Yet SCs had higher actin cytoskeleton organization under all conditions tested, according with our previous data [27]. In particular, the actin cytoskeleton of SCs was elongated fibers when cultured over aligned fibers. Moreover, cells were elongated with their longitudinal axis parallel to the direction of the aligned gelatin nano-fibers, in agreement with previous data [34,35,40,47]. SC seeded on PCL/gelatin random fibers showed cell morphology similar to that on poly-lysine coated coverslips, whereas SC cultured on aligned nano-fibers resulted in aligned morphology, reaching confluence after 9/12 days of culture [40]. Ma *et al*. [35] showed that oriented collagen fibers guide regenerating axons in an oriented way to the distal degenerating nerve stump, maximizing target reinnervation [35]. Finally, aligned nanofibers act as a potential guidance cue by enhancing the alignment of actin filaments, suggesting that these scaffolds would be useful in directing SCs for peripheral nerve regeneration [47]. The data suggest that the organization of SCs in aligned bands mimics the band of Bungner formation and thus leads to improved nerve regeneration and functional recovery *in vivo* [2,3,30,40].

According to adhesion assay data, actin staining of SC showed normal spread morphology at all time-points. SCs proliferated more slowly when seeded on aligned fibers than under control conditions or random fibers. When seeded under aligned fibers, SCs are characterized by elongated actin fiber morphology [47], maintaining an organization in aligned bands with their longitudinal axis parallel to the direction of the aligned gelatin nano-fibers.

MTT assay confirmed that Schwann cell were viable on both random and aligned fibers, demonstrating the biocompatibility of gelatin electrospun fibers [27,28]. It has been reported that blending of gelatin with PCL results in better mechanical properties and hydrophilicity, enhancing nanofibers biocompatibility towards SC [40].

### 3.3. Alignment of Gelatin Electrospun Fibers Does Not Affect Neurite Length but Induce Neurites Alignment

50B11 sensory neurons-like cells have been used to study the influence of aligned nano-fibers on axonal growth. Alignment of gelatin electrospun fibers did not affect the 50B11 adhesion and proliferation rate. Moreover, 50B11 cells maintained their ability to differentiate on gelatin electrospun fibers; on aligned nano-fiber, this resulted in neurites alignment in parallel to the fiber direction. DRG neurites cultured on aligned PCL fibers grow parallel to the fibers, increasing their average length under aligned fibers in comparison with the random fibers [46]. We found that different fiber orientation affects 50B11 neurite orientation, but does not affect neurite length. Neurite organization on aligned and random nano-fibers is in accord with previous reports [34,40,46,47,52].

## 4. Experimental Section

### 4.1. Preparation of Gelatin Solution and Nano-Fibers

Gelatin (GL) solutions were prepared as previously described [28]. Briefly, gelatin (type A from porcine skin), (3-Glycidoxypropyl)methyldiethoxysilane (GPTMS) and polyethylene oxide (PEO, *M*_W_ 900 KDa) were supplied by Sigma Aldrich (Saint Louis, MO, USA). Gelatin was dissolved in demineralized water at 50 °C to the desired concentration (15% *w*/*v*); 137 μL GPTMS were added to the solution and mixed for 1 h before spinning (GL_GPTMS). GPTMS was selected as GL crosslinker to increase the water stability of nanofibers (from few hours to up to 14 days) [28]. Compared to other GL crosslinkers, GPTMS was selected in this study since its crosslinking mechanism does not required a further step after fibers formation that could alter the fibers morphology, due to fiber swelling and partial or complete dissolution. A small amount of PEO was added to stabilize the polymer jet and to enhance aligned fibers formation. PEO was dissolved in distilled water to give a 15% *w*/*v* solution. GL/PEO blend with a 90/10 volume ratio was prepared for aligned nano-fiber fabrication (GL/PEO_GPTMS). 

### 4.2. Electrospinning of Randomly Oriented and Aligned GL Based Nano-Fibers

The electrospinning system consists of an isothermal chamber equipped with: a high voltage generator (PS/EL30R01.5-22 Glassman High Voltage, Inc., Hampshire, UK) giving a voltage up to 30 kV and a current up to 1.5 mA with reversible polarity; a volumetric pump (KD 210, KD Scientific, Hollistone, MA, USA); an electrode; a mobile syringe support; a syringe and different collectors. Electrospun scaffolds were prepared using a vertical electrospinning set-up and 2 different collectors were used: a 1.5 mm-thick flat aluminum collector for random fibers preparation, and a cylindrical rotating drum with an 8 cm diameter and a controllable rotating speed up to 3000 rpm for randomly oriented and aligned fibers deposition, respectively. Randomly oriented nano-fibers were obtained using a GL_GPTMS solution. The solution was spun at 50 °C, 30 kV, flow rate 10 μL·min^−1^ and a nozzle-collector distance of 15 cm to yield fibers of 300 nm, as previously reported [28].

Aligned nano-fibers were collected on rotating mandrel. GL/PEO_GPTMS were spun at 50 °C, 30 kV and the flow rate was increased to 17.5 μL·min^−1^ to obtain homogeneous nano-fibers. The influence of mandrel rotating velocity on fiber alignment was measured.

### 4.3. Scanning Electron Microscopy

The morphology of the electrospun matrices was examined in a scanning electron microscope (SEM Philips 525 M, SEMTech Solutions, Amsterdam, The Netherlands) at an acceleration voltage of 15 kV. The fiber samples were sputter-coated with gold. A magnification of 6000 was selected for 50 μm square fields, allowing fiber distribution to be recorded.

Pore and fiber diameters were quantified by analyzing SEM micrographs with ImageJ software (National Institutes of Health, Bethesda, MD, USA), as previously reported [28,52]. For each fiber type, 3 images from 3 different samples were examined, and the diameters recorded as means ± standard error of the mean.

Nano-fiber orientation under different conditions was examined with a 2D Fast Fourier Transform (FFT) ImageJ processing tool. The applied processing tool shows graphical peaks indicating predominant fiber orientation angles [53,54]

### 4.4. Cell Culture

RT4-D6P2T cells and primary Schwann Cells (SC) were grown on monolayers at 37 °C in a humidified air atmosphere with 5% CO_2_. RT4-D6P2T cells purchased from ATCC (American Type Culture Collection, Manassas, VA, USA 20110-2209) were grown in complete high glucose Dulbecco’s modified Eagle’s medium (DMEM, Invitrogen, Waltham, MA, USA), as per the ATCC protocol.

SCs for primary culture were isolated from the sciatic nerves of adult female Wistar rats (Charles River Laboratories, Milan, Italy) weighing 190–220 g. All procedures were performed in accordance with the Ethics Committee and the European Communities Council Directive of 24 November 1986 (86/609/EEC). Adequate measures were taken to minimize pain and discomfort, taking human endpoints for animal suffering and distress into account. The sciatic nerves were isolated, cut into 3 mm section and incubated at 37 °C in air plus 5% CO_2_ in a complete medium consisting of low glucose DMEM (Gibco, Waltham, MA, USA) supplemented with 100 units·mL^−1^ penicillin, 0.1 mg·mL^−1^ streptomycin, 1 mM sodium pyruvate, 2 mM l-glutamine, 10% heat-inactivated fetal bovine serum (FBS, Invitrogen), 63 ng/mL glial growth factor (GGF, R&D Systems, Minneapolis, MN, USA), and 10 µM forskolin (Sigma, Saint Louis, MO, USA). The medium was changed every 3 days. After 2 weeks, 2 mL digestion solution, consisting of 0.6 mg/mL collagenase type IV (Sigma) and 0.5 mg/mL dispase (Invitrogen) diluted in low glucose complete medium, was added. After 24 h, the incubated nerve segments were transferred to a 50 mL tube and suspended in 5 mL low glucose complete medium. The cell suspension was filtered through a 70 μm strainer (BD Biosciences, San Jose, CA, USA), centrifuged at 900 rpm for 5 min, resuspended in 10 mL of complete SC medium and seeded on poly-l-lysine (PLL, Sigma) coated plates. To remove contaminating fibroblasts, the confluent SCs were immunodepleted. Briefly, the confluent SCs were trypsinized and resuspended in 500 μL low glucose complete medium containing mouse anti-rat Thy1.1 antibody diluted 1:500 (Serotec, MCA04G) and incubated for 10 min at 37 °C. Fresh rabbit complement (250 μL, Cederlane Labs, Burlington, ON, Canada) was added and incubated for 30 min at 37 °C. The reaction was blocked by adding 10 mL low glucose complete medium and the mixture spun for 5 min at 900 rpm. The pellet was resuspended in low glucose complete medium and seeded on PLL-coated plates. Confluent cells were harvested twice a week by trypsinization and seeded at the desired dilution on new plates.

50B11 cells, a gift from Dr. Ahmet Hoke [55], were grown on monolayers at 37 °C in a humidified air atmosphere with 5% CO_2_ in Neurobasal medium (Life Technologies, Gibco) supplemented with 10% FBS (Sigma-Aldrich, Saint Louis, MO, USA), 2% B27 (Life Technologies), 0.22% glucose (Sigma) and 0.2 mM glutamine (Sigma-Aldrich). Before cell seeding, fiber samples were sterilized overnight (O/N) by exposure to UV irradiation (wavelength 254 nm, UV lamp Technoscientific Co., Tokyo, Japan) and incubated in complete DMEM.

### 4.5. Adhesion Assay

RT4-D6P2T and primary SC were seeded in complete DMEM at 4000 or 8000 cells/cm^2^ on both PLL- (control condition) and gelatin-fiber coated coverslips. After 3, 6 or 24 h, the culture medium was removed and the substrates with attached cells were rinsed with PBS containing Ca^2+^ and Mg^2+^ before being fixed by incubation with 4% paraformaldehyde (PFA, Sigma-Aldrich). After 20 min, the PFA solution was removed and the samples rinsed with PBS containing Ca^2+^ and Mg^2+^. The cells were permeabilized with 0.1% Triton X-100 diluted in PBS for 10 min and blocking solution (normal goat serum, NGS, diluted 1:100 in PBS DAKO X0907) was added for 1 h at room temperature. The cells were stained by O/N incubation with anti-vinculin rabbit polyclonal antibody (diluted 1:600 in PBS, Sigma), followed by 1 h incubation with FITC-conjugated phalloidin (diluted 1:1000 in blocking solution, Millipore, Billerica, MA, USA) at room temperature (diluted 1:1000 in PBS, Millipore) and goat-anti rabbit IgG (H + L) AlexaFluor488 (diluted 1:200 in PBS, Invitrogen). Nuclei were stained with 4,6-diamidino-2-phenylindole (DAPI, Sigma) diluted 1:1000 in PBS.

The cells were photographed under an inverted fluorescence microscope Nikon Eclipse 80i equipped with a Nikon ECLIPSE 80i camera using Image-Pro Plus 6.0 (Media Cybernetics, Silver Spring, MD, USA). Cell numbers were calculated using ImageJ software, averaged and expressed as the number of adherent cells/mm^2^ ± standard error of the mean (SEM).

### 4.6. Proliferation

RT4-D6P2T and primary SC were seeded in complete DMEM at 1000 or 2000 cells/cm^2^ on both PLL- (control) and gelatin fiber-coated coverslips. After 1, 3, 5, and 7 days, the cells were fixed, stained, photographed and counted as described above (Section 4.5). The number of cells counted for each assay was averaged and expressed as cells/mm^2^ ± standard error of the mean (SEM).

### 4.7. 3-(4,5-Dimethylthiazol-2-yl)-2,5-diphenyltetrazoliumbromide (MTT) Assay

Potential biomaterial cytotoxicity was evaluated by the MTT assay. RT4-D6P2T and primary SC were plated in 0.2 mL of DMEM containing 10% FBS on both PLL- (control) and gelatin random or aligned nano-fibers (experimental group) coated 96-well tissue culture plate. In order to quantify the cell number serial dilution was performed by plating 1 × 10^3^, 2 × 10^3^, 4 × 10^3^, 8 × 10^3^, 1.6 × 10^4^, 3.2 × 10^4^, and 6.4 × 10^4^ per well. After a 1, 3, 5 and 7 day incubation, 10 μL MTT substrate (Sigma, 5 mg/mL in phosphate buffered saline) was added, and the cells incubated at 37 °C for 4 h. The MTT solution was removed and cells washed twice with 0.1 mL of PBS. 0.1 mL of dimethyl sulfoxide (DMSO; Sigma) was added to each well to dissolve the formazan. Spectrophotometric absorbance was measured at 570 nm wavelength, using DMSO as the blank. Each assay was performed in triplicate.

### 4.8. 50B11 Differentiation

Cells were plated at low densities optimal for visualizing individual neurite growth on both PLL- (control) and gelatin fiber-coated coverslips. Twenty-four hours after plating, the cells were differentiated by adding forskolin (Sigma-Aldrich, 75 μM) to the culture medium. Based on previous observations [55,56], neuronal phenotype was most stable between 20 and 36 h post-forskolin treatment. Twenty-four hours after differentiation, cells were fixed, stained and photographed as described in (Section 4.5), using β-tubulin mouse mAb (diluted 1:100, in PBS, Sigma) and goat-anti-mouse IgG (H + L) AlexaFluor488 (diluted 1:200 in PBS, Invitrogen). Nuclei were stained with DAPI (diluted 1:1000 in PBS, Sigma).

Cell number and axon lengths were measured using *ImageJ* software as described elsewhere [57,58]. The cells counted for each assay were averaged and expressed as cells/mm^2^ ± standard error of the mean (SEM). Axon length was expressed in μm ± standard error of the mean (SEM).

### 4.9. Confocal Microscopy

Samples were observed with a Nikon Eclipse E800 epifluorescence microscope under appropriate filters and a Leica TCS SP5 confocal laser scanning microscope (Leica, Mannheim, Germany) using a 40× Plan-NEOFLUAR objective (numerical aperture (NA) = 1.25) or 63× Plan-NEOFLUAR objective (numerical aperture (NA) = 1.40). High-resolution images were acquired (1024 × 1024 pixels) at 100 Hz.

### 4.10. Statistics

The experiments were repeated 3 times independently and included 3 sets of samples. Each set included 3 random nano-fiber matrix-, 3 align nano-fiber matrix- and 3 control PLL-coated coverslips. The data are expressed as mean ± standard error of the mean (SEM). GraphPad Prism^®^ software was used for single-factor analysis of variance (ANOVA). Values of * *p* < 0.05, ** *p* < 0.01, *** *p* < 0.001 were considered statistically significant.

## 5. Conclusions

The results of the present study show that electrospinning technique can be used to prepare aligned nano-fibers. The alignment nano-fibers induces both primary SC and RT4-D6P2T SC line adhesion and growth in aligned bands with their longitudinal axis parallel to the direction of the fibers compared to control condition and random nano-fibers substrate. Moreover, SCs proliferate more slowly when seeded on aligned fibers than random fibers. Finally, neurite growth of 50B11 neuron-like differentiated cells runs parallel to the aligned fibers.

Our data suggest that: (i) gelatin is a good biomaterial for SCs (both primary SC and RT4-D6P2T SC line) and neurons viability; (ii) the topography of electrospun gelatin fibers can be adjusted to modulate SCs organization and proliferation and axon growth; and (iii) aligned nano-fibers may be promising fillers for the design of artificial organs for peripheral nerve repair. Thus gelatin fibers may be used to induce a parallel orientation and growth of axons and Schwann cells *in vivo* to stimulate bands of Bungner formation resulting in an enhancement of peripheral nerve regeneration.
